# The Crucial Role of Eosinophils in the Life Cycle, Radiographical Architecture, and Risk of Recurrence of Chronic Subdural Hematomas

**DOI:** 10.1089/neur.2020.0036

**Published:** 2021-02-08

**Authors:** Benjamin Davidson, Karl Narvacan, David G. Munoz, Fabio Rotondo, Kalman Kovacs, Stanley Zhang, Michael D. Cusimano

**Affiliations:** ^1^ Division of Neurosurgery, Department of Surgery, University of Toronto, Toronto, Ontario, Canada.; ^2^ Division of Pathology, Department of Laboratory Medicine, University of Toronto, Toronto, Ontario, Canada.; ^3^ Injury Prevention Research Office, Division of Neurosurgery, St. Michael's Hospital, University of Toronto, Toronto, Ontario, Canada.; ^4^ Dalla Lana School of Public Health, University of Toronto, Toronto, Ontario, Canada.

**Keywords:** computed tomography, eosinophil, prediction, recurrence, subdural hematoma

## Abstract

Chronic subdural hematomas (CSDHs) are a common neurological condition, whose incidence is expected to increase with an aging population. Although surgical evacuation is the mainstay of treatment, it results in a recurrence requiring reoperation (RrR) in 3–30% of cases. Recurrence is thought to be driven by a combination of inflammatory and angiogenic processes occurring within the CSDH outer membrane. Pathological specimens of 72 primary CSDHs were examined for eosinophilic infiltrate. For each case, the pre-operative computed tomography (CT) scan was graded according to the Nakaguchi grading scheme as homogeneous, laminar, separated, or trabecular. Rate of RrR was compared based on eosinophilic infiltrate and CT grade. A dense eosinophilic infiltrate was observed in 22% of specimens. The rate of RrR among specimens with a dense eosinophilic infiltrate was 0%, whereas it was 14.3% among specimens without a dense eosinophilic infiltrate. Incidence among homogeneous, laminar, separated, and trabecular CT subtypes was 4%, 27%, 58%, and 24%, respectively. A dense eosinophilic infiltrate found within the outer membrane of a CSDH may be a marker of hematoma maturation, signaling a transition toward healing and fibrosis, and a lower risk of RrR.

## Introduction

A chronic subdural hematoma (CSDH) is a collection of blood, layered between the surface of the brain and dura.^1^ Incidence of CSDH is ∼8–14 per 100,000 person-years,^2–4^ with the mean age of presentation being 76 years.^5^ With the population >65 years of age expected to double between 2010 and 2050, the disease burden of CSDH is anticipated to increase drastically.^6^

Despite the fact that evacuation of a CSDH is a relatively common and routine neurosurgical procedure, recurrence requiring reoperation (RrR) has been reported in 3–30% of cases, with most in the 20% range.^7–10^ Over the past several decades, numerous studies have sought to identify factors associated with recurrence, in hopes of predicting which patients should be monitored more closely in the post-operative period. Although various factors have been associated with RrR, including post-operative hematoma volume,^11^ imaging appearance,^8,11^ and concentration of angiogenic factors,^12–15^ there remains considerable uncertainty regarding the prediction of RrR due to variability between studies.

A central issue that hinders the study of RrR is a lack of understanding concerning the typical life cycle of a CSDH. An early theory regarding the formation of CSDHs stated that after a traumatic brain injury (TBI), rupture of a bridging vein results in slow venous hemorrhage into the subdural space.^16^ However, this theory had several flaws, including the fact that CSDHs often take weeks to materialize after a TBI,^17^ a far slower time course than even the slowest of venous hemorrhages.^18^ Whether or not a small venous bleed is the instigating event, there is general agreement that a traumatic process sets off a cascade of events, including fibroblastic proliferation of the inner dural border cells, inflammation, angiogenesis, recurrent microhemorrhage, hyperfibrinolysis, and exudation.^18^

At the individual patient level, the most direct insight into the life cycle of a CSDH is the computed tomography (CT) scan, routinely obtained on all CSDH patients as part of the pre-operative workup. A small number of studies have attempted to characterize the longitudinal progression of CSDHs based on their CT appearance.^7,8,19^ Although no classification system has perfectly accounted for the vast variability between hematomas, perhaps the most widely used and accepted is that proposed by Nakaguchi and colleagues.^8,11^ They described CSDHs as progressing from a homogeneous type (which progress from hypo- to hyperdense), to a laminar type (with an inner hyperdense layer thought to reflect recent microhemorrhage), to a separated type (with a clearly distinguishable hyper- and hypodense component), and, finally, to a trabecular type (with numerous septations). Nakaguchi and colleagues' original article and subsequent studies presented evidence that the laminar and separated types of hematoma are most likely to recur, whereas the trabecular type is least likely to recur after surgical evacuation.^8,11^ In the life cycle of a CSDH, a trabecular appearance on CT may indicate that the hematoma is beginning to resolve.^8,11,19^

In the process of unraveling the life cycle of the CSDH, a particularly elusive phenomenon has been the presence of eosinophils within the outer CSDH membrane. Eosinophils, which make up 1% of white blood cells, are not endogenous to the brain and yet are present in up to 50% of all CSDH surgical specimens.^20,21^ Although best known for their pathological role in asthma and other allergic processes, eosinophils generally play a vital role in combating various types of pathogens and have been suggested to play a role in suppressing some types of cancer.^22^ Eosinophils act as sentinels or monitors of the local milieu and, in response to certain cytokines, can initiate processes of inflammation or repair.^23^ The presence of eosinophils within the outer membrane of a CSDH has never been studied in relation to CT appearance or, more importantly, RrR.

In this study, we sought to determine the relationship of eosinophils to the CT appearance and risk of recurrence in CSDHs. Given that inflammation has been implicated in the growth and persistence of CSDHs, we hypothesized that the presence of eosinophils would be most strongly associated with laminar and separated CT appearances, as well as an increased risk of RrR.

## Methods

**FIG. 1.**
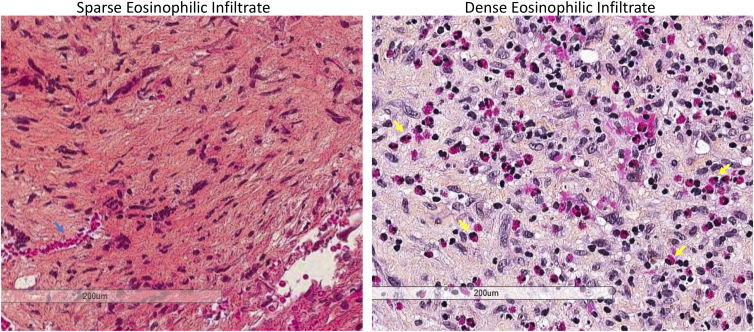
Representative slides from specimens graded as having sparse eosinophilic infiltrate (left) and dense eosinophilic infiltrate (right). Blue arrow on left image indicates a blood vessel. Yellow arrows on right image indicate eosinophils.

**FIG. 2.**
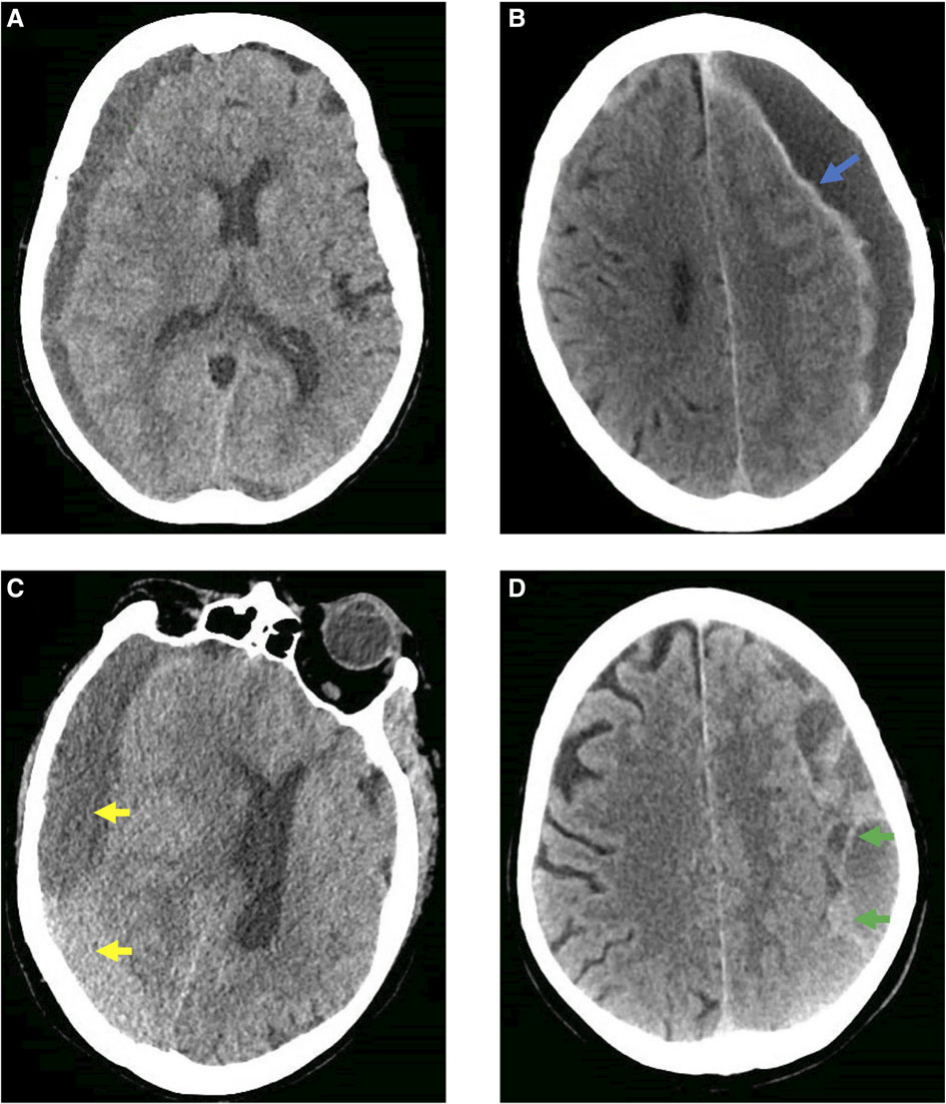
Illustration of imaging classification scheme introduced by Nakaguchi and colleagues.^8^ (**A**) Homogeneous type. (**B**) Laminar type, characterized by hyperdense layering along the inner membrane of the subdural (hyperdense layering indicated by blue arrow). (**C**) Separated type, wherein two distinct regions of hematoma are appreciated (two regions indicated with yellow arrows). (**D**) Trabecular: numerous membranes visible within the subdural space (a membrane indicated with green arrows).

After approval from the Research Ethics Board of St Michael's Hospital (Toronto, Ontario, Canada), 72 surgically removed CSDH (operated between 2016 and 2020) outer membrane specimens were analyzed histologically. All specimens were stained with hematoxylin and eosin (H&E) and examined using light microscopy. They were graded for eosinophilic infiltration as 0 (absent), 1 (sparse), 2 (moderate), or 3 (dense). Eosinophils were graded by two physicians (B.D. and K.N.) blinded to clinical details. For the purposes of analysis, specimens graded as 0 or 1 were considered together as “sparse eosinophilic infiltrate,” and specimens graded as 2 or 3 were considered together as “dense eosinophilic infiltrate” (Fig. 1). The pre-operative CT appearance of each CSDH was evaluated and classified as homogeneous, laminar, loculated, or separated as outlined by Nakaguchi and colleagues^8^ (Fig. 2) by a single physician (B.D.) who was blinded to clinical outcomes. This study was conducted in accordance with the Strengthening the Reporting of Observational Studies in Epidemiology (STROBE) guidelines (Supplementary Material).

## Results

Clinical descriptions of CSDH cases are presented in Table 1. Average age (years) was 73.4 years. CSDH evacuations were performed with minicraniotomy on 48 of 72 specimens, whereas the remaining 24 of 72 were evacuated using burr hole(s) (1 of 24 cases used two burr holes). In all cases, a subdural drain was left in place for 1–3 days after the procedure, with the exception of 1 case where no drain was inserted and another where two drains were inserted. The overall RrR was 11.1%.

**Table 1.** Clinical Description of CSDH Specimens

	No. of patients	No. of hematomas	Age	M:F	Procedure	RrR
Primary specimens (*n* = 72)	68	72	73.4 (11.0)	1.8:1.0	Single burr hole: 48 Minicraniotomy: 24	11.1%

Brackets indicate standard deviation.

CSDH, chronic subdural hematoma; RrR, recurrence requiring reoperation; M, male; F, female.

### Recurrence requiring reoperation based on eosinophilic infiltration and imaging classification

**FIG. 3.**
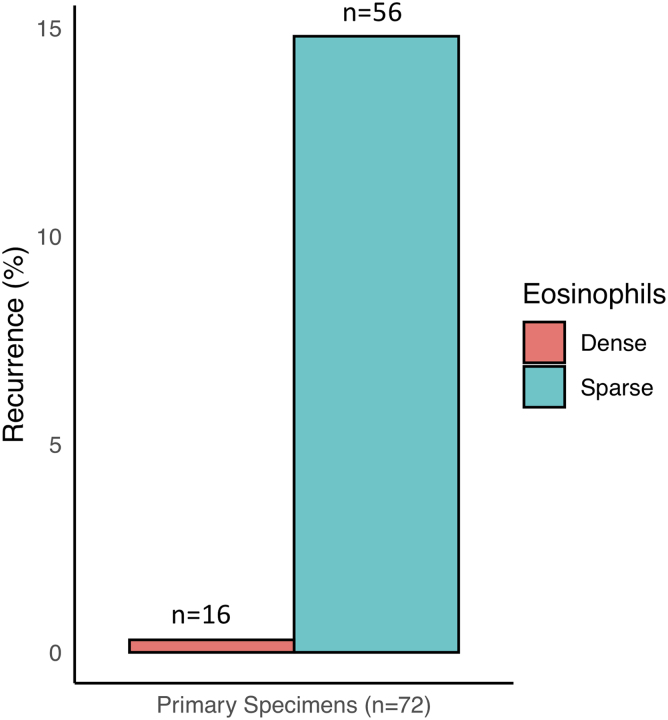
Recurrence requiring reoperation (RrR) in primary specimens, categorized by eosinophil concentration.

**FIG. 4.**
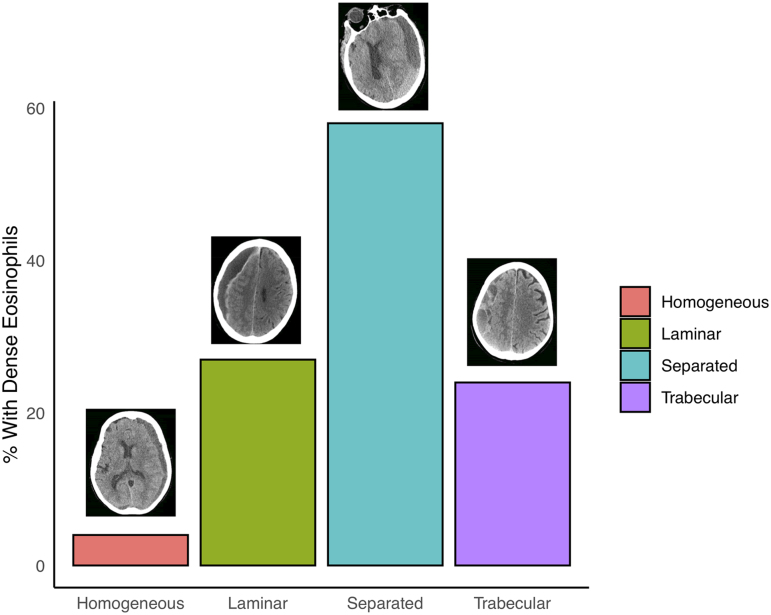
Percentage of primary specimens with dense eosinophilic infiltrate, sorted by CT subtype. CT, computed tomography.

A dense eosinophilic infiltrate was observed in 22.2% of cases; the remainder either had no eosinophil infiltrate at all or a sparse infiltrate. The RrR was 14.3% in specimens with sparse eosinophilic infiltrate and 0% in specimens with dense eosinophilic infiltrate (Fig. 3). Occurrence of each hematoma type was: homogeneous (38.9%), laminar (20.8%), separated (16.7%), and trabecular (23.6%). The percentage of each of these imaging types found to have a dense eosinophilic infiltrate was 4%, 27%, 58%, and 24%, respectively (note that these percentages do not sum to 100, because they represent a proportion in each imaging type; Fig. 4). The RrR among homogeneous, laminar, separated, and trabecular hematomas was 14%, 20%, 0%, and 6%, respectively.

## Discussion

Despite the rising incidence of CSDH, there remains considerable uncertainty regarding the predictors of RrR after surgical evacuation. The difficulty in identifying predictors of RrR may have to do with the fact that CSDHs can present as various points in their life cycle. Based on the results of this study, we argue that eosinophils are a robust marker of a CSDH's life-cyle stage and thus can help predict the risk of RrR. The most remarkable finding of our study was that the incidence of RrR in primary CSDH specimens *with* a dense eosinophilic infiltrate was 0% (compared to 14.3% in specimens *with a sparse or absent* eosinophilic infiltrate). Although eosinophilic infiltrate was observed in all CT types, it was least commonly associated with homogeneous CSDHs. The fact that eosinophils are present only in a subset of CSDHs suggests that this leukocyte may provide some insight into the life-cycle stage or trajectory of a CSDH.

As far back as 1857, Virchow was the first to associate CSDHs with inflammation, calling the pathology “pachymeningitis haemorrhagica interna.”^24^ The inflammatory process begins with the inner layer of dura, which is lined with a specialized type of connective tissue cell, called the “dural border cells.”^18^ These cells, when injured, proliferate and release inflammatory mediators, intended to repair the injury, but in a subset of patients/situations, they trigger a prolonged course of inflammation and the development of a CSDH.^1^ In those cases, dural border cells continue to proliferate, eventually forming an inner and outer membrane encapsulating a CSDH. Whereas the inner membrane is relatively non-functional, the outer membrane contains neutrophils, lymphocytes, inflammatory/angiogenic markers, and newly forming blood vessels. These blood vessels, thought to potentially be recruited from terminal branches of the middle meningeal artery, form in a haphazard way and lack normal basement membranes and tight junctions.^25,26^ Therefore, it has been hypothesized that these vessels allow for a continuous exudative “leak” into the subdural space, resulting in growth. The outer membrane is also thought to provide fibrinolytic agents, which maintain liquidity of the CSDH, such as tissue plasminogen activator and thrombomodulin.^27,28^

In addition to the inner and outer membranes described above, over time some CSDHs develop membranes within their cavities. Although the exact mechanism driving this process is not known, it fits with the concept of repair and remodeling occurring in the setting of prolonged inflammation. As these “intrahematoma” membranes form, they go through a process of maturation. Early membranes are relatively immature, with newly formed blood vessels and an increased risk of microhemorrhage. These membranes may be observed in the laminar and separated types of CSDH. Over time, these membranes seem to mature and thicken, into what is noted in trabecular hematomas, with a much lower risk of hemorrhage.^8,11^

Based on the findings of this study, eosinophils may gradually accumulate as a CSDH progresses from the homogeneous to separated stage. This is consistent with two previous studies which analyzed eosinophils in CSDH.^20,29^ Both studies reported that eosinophils do not appear within CSDHs at an acute or subacute stage, but rather appear to gradually accumulate as CSDHs mature. Coupled with the findings of this study, eosinophils may play a pivotal role in the reparative/resorptive process of fibrosis and membranization, which becomes apparent during the trabecular stage. If a separated CSDH has not yet been infiltrated with eosinophils, it may suggest that this hematoma is still undergoing early inflammation/angiogenesis and is not yet at the stage of developing mature organizing membranes, and therefore more at risk of recurrence with a simple burr-hole drainage procedure alone.

The advantage of studying eosinophils in the context of CSDH is that eosinophilic infiltrate can be easily reported without any expensive stains or rare tests. With a simple H&E stain, a pathologist in any country can readily detect the presence or absence of dense eosinophilic infiltrate, making this an inexpensive and practical test to implement. Knowing the life cycle of the eosinophilic/inflammatory life cycle may also lead to new, or earlier, treatment options for clinicians.

### Limitations

Despite being the largest series to study eosinophilic infiltration, studies with larger sample sizes will be required to confirm that a dense eosinophilic infiltrate predicts a lack of RrR. CSDH specimens were not obtained from every CSDH evacuation performed at our institution. They were obtained at the surgeon's discretion, and some were more likely to collect a specimen than others, so our results may be subject to surgeon bias. However, patients with CSDH are referred to the “on-call” surgeon based on relatively random on-call schedules, so we are confident the impact of this would be minimal. It can be technically difficult to collect a specimen during burr-hole evacuation, thus biasing the sample toward CSDHs treated with a craniotomy. Fortunately, despite a craniotomy being more conducive to collecting a biopsy, 24 of 72 specimens in this sample were obtained through a burr hole, reducing the chance of these findings being the result of a sampling error. Although in this study we have characterized the presence or absence of eosinophils within the outer membranes of CSDHs, we were not able to comment on the activity of these eosinophils. Eosinophils are known to be capable of releasing their granules, effecting widespread cascades involving inflammation and repair. Further studies, using electron microscopy or measuring downstream mediators released by eosinophils, such as transforming growth factor beta 1, will help clarify this point.

## Conclusions

Presence of a dense eosinophilic infiltrate was associated with a reduced risk of recurrence after surgery for CSDH. Dense eosinophilic infiltrate was most commonly associated with the laminar and separated types of CSDH thought to be a late stage of maturation. To this end, eosinophils may play a role in inducing membrane formation, repair, and fibrosis and therefore reducing the subsequent risk of recurrence of CSDHs. These results will be important to international public health given the incidence of CSDH in every country. Detectable on standard H&E staining, the presence or absence of eosinophils can serve as an affordable, easily accessible, and easily measured biomarker for CSDHs, helping to identify patients in low-, middle-, and high-income countries, who should be monitored more closely after surgery.

## Supplementary Material

Supplemental data

## Supplementary Material

STROBE Checklist

## Abbreviations Used

CSDH: chronic subdural hematoma
CT: computed tomography
H&E: hematoxylin and eosin
RrR: recurrence requiring reoperation
