# Supplementary material for: The Crucial Role of Eosinophils in the Life Cycle, Radiographical Architecture, and Risk of Recurrence of Chronic Subdural Hematomas
Source: Neurotrauma Rep. 2021 Feb 8;2(1):76–83. doi: 10.1089/neur.2020.0036 (PMC8240825; doi:10.1089/neur.2020.0036)
Supplement: Supplemental data [file Supp_Data.docx]

**Supplementary Materials**

STROBE Statement—checklist of items that should be included in reports of observational studies

|  | Item No. | Recommendation | Page  No. | Relevant text from manuscript |
| --- | --- | --- | --- | --- |
| **Title and abstract** | 1 | (*a*) Indicate the study’s design with a commonly used term in the title or the abstract | N/A | N/A |
|  |  | (*b*) Provide in the abstract an informative and balanced summary of what was done and what was found | 1 | See Methods/Findings/Interpretation of abstract |
| Introduction | | | |  |
| Background/rationale | 2 | Explain the scientific background and rationale for the investigation being reported | 3 | See introduction |
| Objectives | 3 | State specific objectives, including any prespecified hypotheses | 3 | Page 3: we hypothesized that the presence of eosinophils would be most strongly associated with laminar and separated CT appearances, as well as an increased risk of RrR. |
| Methods | | | |  |
| Study design | 4 | Present key elements of study design early in the paper | 3 | See methods section |
| Setting | 5 | Describe the setting, locations, and relevant dates, including periods of recruitment, exposure, follow-up, and data collection | 3 | See methods section |
| Participants | 6 | (*a*) *Cohort study*—Give the eligibility criteria, and the sources and methods of selection of participants. Describe methods of follow-up | 3 | 72 surgically removed CSDH (operated between 2016-2020) outer membrane specimens were analyzed histologically. |
|  |  | (*b*) *Cohort study*—For matched studies, give matching criteria and number of exposed and unexposed | N/A | N/A – no matching groups were used. |
| Variables | 7 | Clearly define all outcomes, exposures, predictors, potential confounders, and effect modifiers. Give diagnostic criteria, if applicable | 3 | Primary outcome: Risk of recurrence requiring reoperation. Predictors: eosinophilic infiltration, CT-subtype |
| Data sources/ measurement | 8* | For each variable of interest, give sources of data and details of methods of assessment (measurement). Describe comparability of assessment methods if there is more than one group | 3 | See methods section |
| Bias | 9 | Describe any efforts to address potential sources of bias | 3 | Methods: Two blinded raters of eosinophilic concentration |
| Study size | 10 | Explain how the study size was arrived at | 3 | All available specimens (consecutive) were included. |

| Quantitative variables | 11 | Explain how quantitative variables were handled in the analyses. If applicable, describe which groupings were chosen and why | N/A | No statistical analysis, raw data is presented in figures/tables |
| --- | --- | --- | --- | --- |
| Statistical methods | 12 | (*a*) Describe all statistical methods, including those used to control for confounding | N/A | No statistical methods were used |
|  |  | (*b*) Describe any methods used to examine subgroups and interactions | N/A | No subgroups were analyzed |
|  |  | (*c*) Explain how missing data were addressed | N/A | There was no missing data |
|  |  | (*d*) *Cohort study*—If applicable, explain how loss to follow-up was addressed | 3 | Last followup assessment was used to determine presence or absence of recurrence |
|  |  | (*e*) Describe any sensitivity analyses | N/A | Not performed |
| Results | | | | |
| Participants | 13* | (a) Report numbers of individuals at each stage of study—eg numbers potentially eligible, examined for eligibility, confirmed eligible, included in the study, completing follow-up, and analysed | 4 | 72 patients |
|  |  | (b) Give reasons for non-participation at each stage | N/A |  |
|  |  | (c) Consider use of a flow diagram | N/A |  |
| Descriptive data | 14* | (a) Give characteristics of study participants (eg demographic, clinical, social) and information on exposures and potential confounders | Page 4/Table 1 | See 1^st^ paragraph of results and Table 1 |
|  |  | (b) Indicate number of participants with missing data for each variable of interest | N/A | No missing data |
|  |  | (c) *Cohort study*—Summarise follow-up time (eg, average and total amount) | N/A | There was no followup involved in this study |
| Outcome data | 15* | *Cohort study*—Report numbers of outcome events or summary measures over time | 4 | Table 1, Figures 3-4 |
|  |  | *Case-control study—*Report numbers in each exposure category, or summary measures of exposure | N/A | No exposure categories |
|  |  | *Cross-sectional study—*Report numbers of outcome events or summary measures | N/A |  |
| Main results | 16 | (*a*) Give unadjusted estimates and, if applicable, confounder-adjusted estimates and their precision (eg, 95% confidence interval). Make clear which confounders were adjusted for and why they were included | N/A |  |
|  |  | (*b*) Report category boundaries when continuous variables were categorized | N/A |  |
|  |  | (*c*) If relevant, consider translating estimates of relative risk into absolute risk for a meaningful time period | N/A |  |

Continued on next page

| Other analyses | 17 | Report other analyses done—eg analyses of subgroups and interactions, and sensitivity analyses | N/A | All data is presented |
| --- | --- | --- | --- | --- |
| Discussion | | | | |
| Key results | 18 | Summarise key results with reference to study objectives | 4 | The most remarkable finding of our study was that the incidence of RrR in primary CSDH specimens *with* a dense eosinophilic infiltrate was 0% (compared to 14.3% in specimens *with a sparse or absent* eosinophilic infiltrate). Although eosinophilic infiltrate was observed in all CT types, it was least commonly associated with homogeneous CSDH’s. The fact that eosinophils are present only in a subset of CSDH’s suggests that this leukocyte may provide some insight into the lifecycle stage or trajectory of a CSDH. |
| Limitations | 19 | Discuss limitations of the study, taking into account sources of potential bias or imprecision. Discuss both direction and magnitude of any potential bias | 5 | Despite being the largest series to study eosinophilic infiltration, studies with larger sample sizes will be required to confirm that a dense eosinophilic infiltrate predicts a lack of RrR. CSDH specimens were not obtained from every CSDH evacuation performed at our institution. They were obtained at the surgeon’s discretion, and some were more likely to collect a specimen than others, so our results may be subject to surgeon bias. However, patients with CSDH are referred to the “on-call” surgeon based on relatively random on-call schedules, so we confident the impact of this would be minimal. However, it can be technically difficult to collect a specimen during burr-hole evacuation, thus biasing the sample towards CSDH’s treated with a craniotomy. Although in this study we have characterized the presence or absence of eosinophils within the outer membranes of CSDH’s, we were not able to comment on the activity of these eosinophils. Eosinophils are known to be capable of releasing their granules, effecting widespread cascades involving inflammation and repair. Further studies using electron microscopy or measuring downstream mediators released by eosinophils, such as TGF-B1 will help clarify this point. |
| Interpretation | 20 | Give a cautious overall interpretation of results considering objectives, limitations, multiplicity of analyses, results from similar studies, and other relevant evidence | 5 | See limitations.  Conclusion: The presence of a dense eosinophilic infiltrate was associated with a reduced risk of recurrence after surgery for CSDH. Dense eosinophilic infiltrate was most commonly associated with the laminar and separated types of CSDH thought to be a late stage of maturation. To this end, it is thought that eosinophils may play a role in inducing membrane formation, repair, and fibrosis, and the subsequent risk of recurrence of CSDH.. These results will be important to international public health given the incidence of CSDH in every country. Detectable on standard hematoxylin and eosin staining, the presence or absence of eosinophils can serve as an affordable, easily accessible and easily measured biomarker for CSDH’s, helping to identify patients in low, middle and high income countries, who should be monitored more closely after surgery. |
| Generalisability | 21 | Discuss the generalisability (external validity) of the study results | 5 | See limitations |
| Other information | |  | | |
| Funding | 22 | Give the source of funding and the role of the funders for the present study and, if applicable, for the original study on which the present article is based | Title Page | This work was funded in part by the Codman Neurotrauma Research Grant in association with the American Association Neurological Surgeons, as well as the AFP Innovation Fund and health research funds from PSI Foundation.  Authors are also grateful for the continued support from the Jarislowsky and Lloyd Carr-Harris Foundations. |
